# Effective Single-Mode Methodology for Strongly Coupled Multimode Molecular-Plasmon Nanosystems

**DOI:** 10.1021/acs.nanolett.3c00735

**Published:** 2023-05-23

**Authors:** Marco Romanelli, Rosario Roberto Riso, Tor S. Haugland, Enrico Ronca, Stefano Corni, Henrik Koch

**Affiliations:** † Department of Chemical Sciences, University of Padova, via Marzolo 1, 35131 Padova, Italy; ‡ Department of Chemistry, Norwegian University of Science and Technology, 7491 Trondheim, Norway; § Department of Chemistry, Biology and Biotechnology, University of Perugia, via Elce di Sotto, 8, 06123 Perugia, Italy; ∥ CNR Institute of Nanoscience, via Campi 213/A, 41125 Modena, Italy; ⊥ Padua Quantum Technologies Research Center, University of Padova, 35131 Padova, Italy; # Scuola Normale Superiore, Piazza dei Cavalieri 7, 56126 Pisa, Italy

**Keywords:** plasmonics, strong coupling, coupled cluster theory

## Abstract

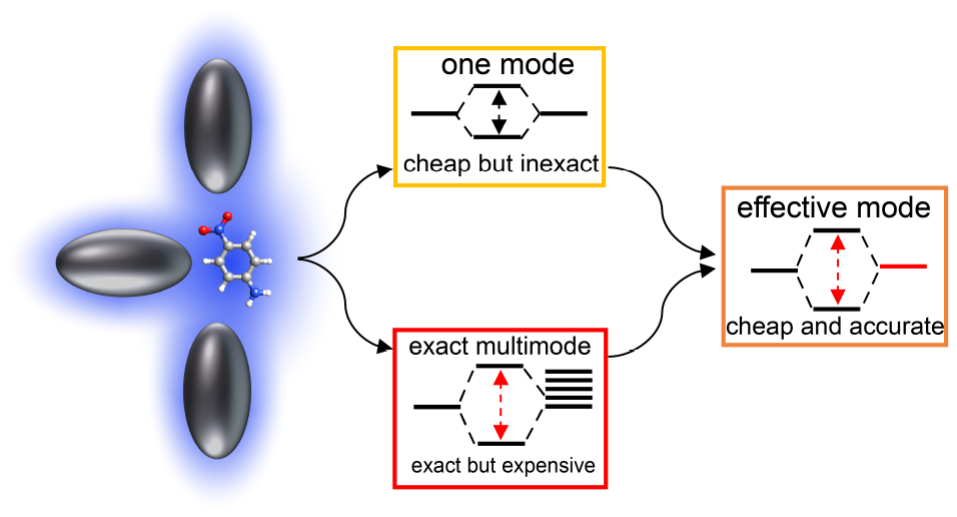

Strong coupling between molecules and quantized fields has emerged as an effective methodology to engineer molecular properties. New hybrid states are formed when molecules interact with quantized fields. Since the properties of these states can be modulated by fine-tuning the field features, an exciting and new side of chemistry can be explored. In particular, significant modifications of the molecular properties can be achieved in plasmonic nanocavities, where the field quantization volume is reduced to subnanometric volumes, thus leading to intriguing applications such as single-molecule imaging and high-resolution spectroscopy. In this work, we focus on phenomena where the simultaneous effects of multiple plasmonic modes are critical. We propose a theoretical methodology to account for many plasmonic modes simultaneously while retaining computational feasibility. Our approach is conceptually simple and allows us to accurately account for the multimode effects and rationalize the nature of the interaction between multiple plasmonic excitations and molecules.

Strong light–matter coupling between molecules and electromagnetic fields leads to the formation of new hybrid states, known as polaritons, where the quantum nature of the electromagnetic field entangles with purely molecular states.^1−8^ The resulting polaritons can display different key features compared to the original states, potentially leading to new chemical/photochemical reactivity,^1,9−15^ energy transfer processes,^16−21^ or relaxation channels,^15,22−24^ among others. While photonic cavities are an obvious choice, other fields, like the ones produced by electronic excitations in plasmonic nanostructure, can also be used to achieve the strong coupling regime. Despite their highly lossy nature, plasmonic nanocavities can confine the electromagnetic fields even down to subnanometric volumes.^25^ The resulting interaction could be instrumental for a wide range of applications, such as sensing,^26−29^ high-resolution spectroscopy,^30−32^ single-molecule imaging,^32−34^ and photocatalysis.^35−39^

Recent works point out that the simultaneous contribution of multiple plasmonic modes, going beyond the simplest dipolar resonances, might be critical for a number of phenomena,^40−43^ e.g., the chiro-optical response of light–matter systems.^44−51^ In such cases, theoretical models that capture multiple plasmon modes simultaneously are of the utmost importance.

Several *ab initio* quantum electrodynamics (QED) methods for strongly coupled systems have been proposed, e.g., quantum electrodynamics density functional theory (QEDFT),^3,52,53^ QED coupled cluster (QED-CC),^54−58^ and quantum electrodynamics full configuration interaction (QED-FCI).^55,59^ Despite its computational affordability, QEDFT inherits the intrinsic problems of exchange and correlation functionals,^60,61^ whereas QED-CC, albeit more accurate, is computationally demanding. The latter method has recently been extended to model quantized plasmonic modes obtained through a polarizable continuum model (PCM)^62^ description of the nanoparticle response (Q-PCM-NP).^63^ In its current implementation, however, QED-CC cannot take into account more than one plasmon mode at a time. Generalization of the original theory to the multimode case will quickly become computationally unfeasible.

In this paper, we couple the existing plasmon QED-CC method to a scheme that captures the main effects of multiple plasmons into a single effective mode. This allows us to retain the same computational cost of a single-mode QED-CC calculation while accounting for the multimode effects.

We first present a formal definition of the effective mode, followed by a numerical example on a test case system. Specifically, the effective mode approach is tested on a system composed of three nanoparticles (NPs) surrounding either a hydrogen or a *para*-nitroaniline (PNA) molecule. For hydrogen, we benchmark the effective mode approach against multimode QED-FCI. At the end, our final considerations and perspectives on the proposed method are given.

In our framework, the nanoparticle (NP) is described using the Drude–Lorentz dielectric function model,^64^ that is
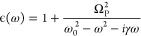
1where Ω_P_ is the bulk plasma frequency; ω_0_ is the natural frequency of the bound oscillator; and γ is the damping rate. Together, these quantities define the nanoparticle material. The technique to quantize the NP linear response through a PCM-based theory has already been reported in a previous work.^63^ In summary, the nanoparticle surface is described as a discretized collection of tesserae, labeled by *j*, each of which can host a variable surface charge representing the NP response to a given external perturbation.^65−67^ The key quantity obtained from the PCM-based quantization scheme is *q*_*pj*_ which can be identified as the transition charge sitting on the *j*th tessera of the NP for a given excited state p. The collection of all the charges for a given *p*-mode represents one possible normal mode of the NP (a plasmon), with frequency ω_*p*_. The detailed theory formulation can be found in the original work^63^ where the above-mentioned quantities are explicitly derived.

On this basis, the Hamiltonian used to describe the interaction between the nanoparticle and the molecule equals
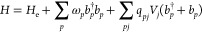
2where *H*_e_ is the standard electronic Hamiltonian;^68^ ω_*p*_ is the frequency of the *p*th nanoparticle mode; and the operators  and *b*_*p*_ create and annihilate plasmonic excitations of frequency ω_*p*_, respectively. The interaction between the molecule and the plasmon is mediated through the bilinear term
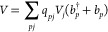
3In eq 3, *V*_*j*_ is the molecular electrostatic potential operator evaluated at the *j*th tessera of the NP, while *q*_*pj*_ is the quantized charge of mode *p* that lies on the *j*th tessera. From eq 3, the plasmon–molecule coupling for a transition going from the molecular state *S*_0_ to *S*_*n*_ and exciting the plasmon mode *p* reads
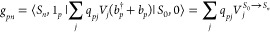
4where  is the potential coming from the *S*_0_ → *S*_*n*_ transition density at the *j*th tessera of the NP surface. The coupling terms in eq 4 are the key quantities for simpler approaches to the strong-coupling regime, such as the Jaynes–Cummings (JC) model.^69^ This is also the starting point of the effective mode derivation presented in this work. Using the full Hamiltonian in eq 2 is indeed computationally expensive because of the elevated number of plasmon modes that need to be considered. For this reason, it is customary to only include one mode in the Hamiltonian. While the single-mode approximation has been used with great success in the past, there are instances where a multimode approach is necessary. One example, for instance, is when multiple plasmonic excitations are almost resonant with the same molecular excitation or, as already discussed previously, when circular dichroism phenomena are studied. To reduce the computational cost while retaining a reasonable accuracy, it would be desirable to define a single effective boson that accounts, on average, for the effect of many modes. In our framework the effective mode will be obtained starting from a multimode JC Hamiltonian.

The generalization of the single-mode JC Hamiltonian to a multimode plasmonic system is
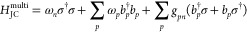
5where ω_*n*_ is the frequency of the *S*_0_ → *S*_*n*_ excitation and σ^†^, σ is the molecular raising and lowering operators.
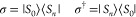
6In our case, ω_*n*_ and *g*_*pn*_ are the excitation energies and the plasmon-mediated transition coupling elements computed using coupled cluster singles and doubles (CCSD) (more details can be found in the SI). Diagonalization of the Hamiltonian in eq 5 yields the mixed plasmonic–molecular wave functions with corresponding energies. We will simply use the term “polaritonic” to generally refer to those hybrid states from now on even though a mixed plasmon–electronic excitation state is properly called plexciton.^63^ In the single mode case, the two eigenstates, typically called lower and upper polaritons (LP, UP), are given by
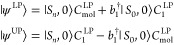
7with  and  being the coefficients of the molecular excited state with no plasmons and the molecular ground state with one plasmonic excitation, respectively. These coefficients also appear in the UP wave function because of the orthogonality constraints.

On the other hand, the eigenfunctions of the Hamiltonian in eq 5 for the multimode case read
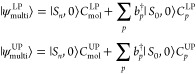
8where the coefficients defining the two polaritonic wave functions do not have to satisfy the strict relation in eq 7. Moreover, they can be rewritten as
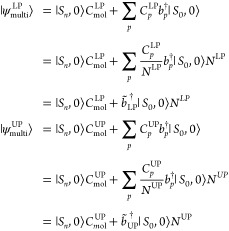
9where index *p* labels the plasmon modes and the normalization factors *N*^LP^ and *N*^UP^ are defined as
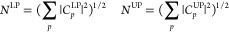
10The effective lower and upper polariton bosons are given by
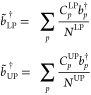
11and they have been introduced to describe the plasmon part of the lower and upper polaritons. The normalization term *N*^LP/UP^ is needed to ensure that the bosons still respect the commutation relations:
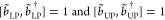
12We point out that, unlike the single-mode case in eq 7, the effective lower and upper polariton boson operators are different from each other. Moreover, the two bosons have a nonzero overlap such that
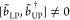
13The effective mode approximation comes into play when we seek a single effective mode  that replaces both  and  such that the energies obtained using the effective upper and lower polaritonic states
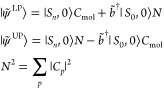
14are as close as possible to the ones obtained using the multimode JC model. We notice that in eq 14 the *C*_*p*_ coefficients are now common to both the lower and upper polariton wave functions. Specifically, they are optimized by minimizing the functional
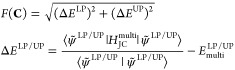
15where  and  are the LP and UP energies obtained by diagonalizing the Hamiltonian in eq 5. The optimal coefficients defining the effective mode are the actual output of that functional minimization and are system specific; that is, the effective mode composition will vary if the molecule and/or the plasmonic system change. Besides this, we note that the two solutions shown in eq 14 resemble the structure of the exact single-mode case in eq 7. Nonetheless, the plasmonic part of the wave function captures the effect of multiple modes at the same time. The procedure described here can easily be generalized to the case of an optical cavity.

Once the effective mode has been defined, the Hamiltonian in eq 2 can be rewritten as
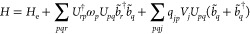
16where  is the effective mode defined in the previous section and the other bosonic operators fulfill
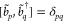
17The two bosonic bases are related by a unitary transformation *U*
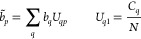
18Truncating the plasmon modes in eq 16 to only include the effective mode , the Hamiltonian reads
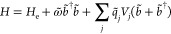
19where the following quantities have been introduced
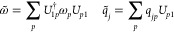
20The quantized charge , of the effective plasmon mode , allows for a direct visualization of the effective mode properties (see Figure 2c).

Starting from the Hamiltonian in eq 19, we can use any single-mode QED method to study the effects of multiple plasmonic modes on molecular properties. In this work, we focus on the QED-CC approach. The QED-CC approach is the natural extension of standard coupled cluster theory to the strong coupling regime. The wave function is parametrized as
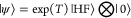
21where |HF⟩ is the reference Slater determinant (usually obtained through a Hartree–Fock like procedure), while |0⟩ denotes the plasmonic vacuum. The cluster operator *T* is defined as
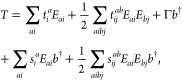
22with each term corresponding to an electron, electron–plasmon, or plasmon excitation. In eq 22, the electronic second quantization formalism has been adopted such that^68^
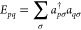
23where  and *a*_*qσ*_ create and annihilate an electron with spin σ in orbitals *p* and *q*, respectively. Following the commonly used notation, we denote the unoccupied HF orbitals with the letters *a*, *b*, *c*..., while for the occupied orbitals we use *i*, *j*, *k*.^68^ Inclusion of the full set of excitations in eq 22 leads to the same results as QED-FCI. In this work we truncate *T* to include up to one plasmon excitation as well as single and double electronic excitations in line with what has been presented in ref (54). The parameters , and Γ are called amplitudes. They are determined solving the projection equations
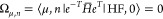
24where μ is an electronic excitation, while *n* is a plasmonic excitation. We adopted the notation
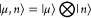
25The  operator is the molecule-plasmon Hamiltonian in eq 19 transformed with a coherent state. This accounts for the polarization of the plasmonic system induced by the molecular charge density in the HF state.
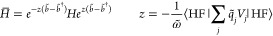
26

The setup we employed to test the effective mode approach consists of three identical ellipsoidal NPs, each one featuring a long-axis length of 6.0 nm and a short-axis length of 2.0 nm. In between the nanoellipses we placed first an H_2_ and later a PNA molecule that are approximately 0.6 nm away from the three structures, as shown in Figure 1. This setup was chosen because it has degenerate (or almost degenerate) plasmon modes with significant coupling to the molecule. Moreover, the plasmon frequencies can easily be modulated, for instance by changing the aspect ratio of the ellipsoidal NPs (see Figure S2 in the SI as an example). Additional details about the computational methodologies can be found in the last section of the SI.^34,70,71^

**Figure 1:**
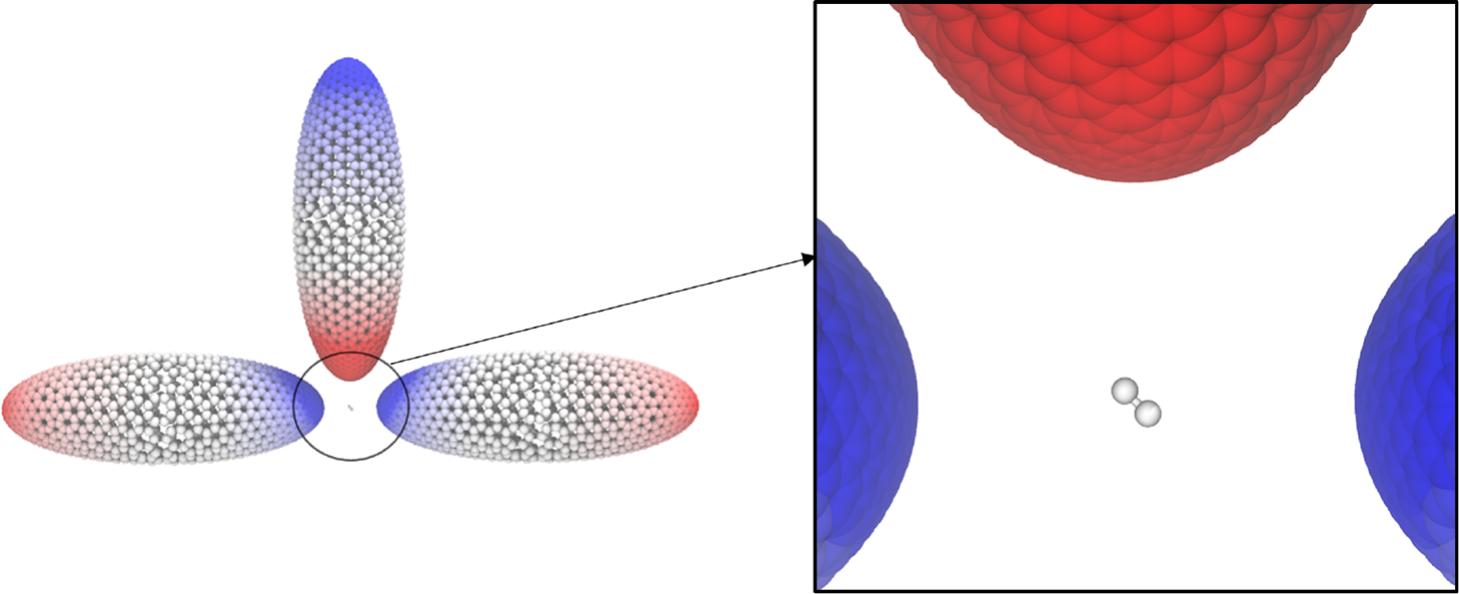
Setup employed to test the effective mode scheme. The plasmonic system consists of 3 ellipsoidal NPs surrounding an H_2_ molecule in the *yz* plane. The beads composing the NPs represent the centroids of each tessera upon surface discretization and host a given quantized charge *q*_*pj*_. The lowest (in energy) plasmon mode is shown. Red beads refer to positive charges, whereas blue ones refer to negative charges. Each NP is ≈0.6 nm far from H_2_.

The NP setup shown in Figure 2 has two almost degenerate low excitations (Figure 2 a and b) at 12.661 and 12.677 eV, whose coupling parameters with the first H_2_ transition are 8.6 and 15.2 meV, respectively. Both excitations will significantly contribute to the effective mode. Specifically, their coefficients in the expansion of the effective mode (see eq 18) are reported in the top part of Figure 2.

**Figure 2:**
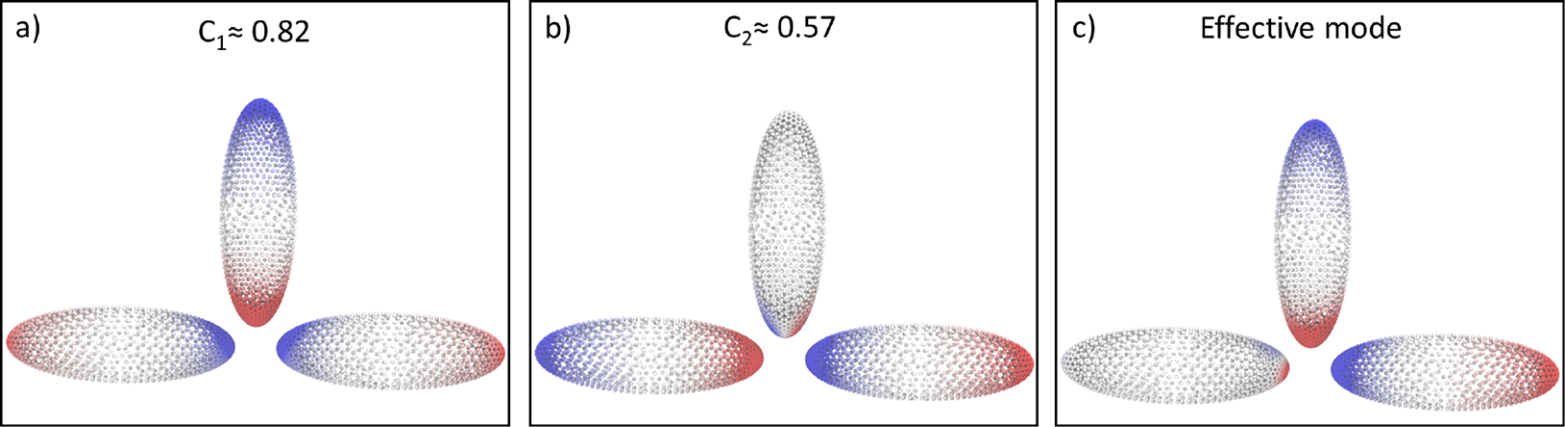
First (a) and second (b) quasi-degenerate plasmon modes of the setup shown in Figure 1. The energy splitting between these two modes is ≈16 meV, and they both significantly couple to the *S*_0_ → *S*_1_ transition of H_2_. Their contribution to the effective mode is shown at the top of the panel. (c) Visualization of the optimized effective mode. Only the two most important modes are reported in panels (a) and (b), but the first 12 modes coupling to the molecular transition contribute to the effective mode optimization.

In Figure 3 we show how the inclusion of multiple plasmon modes affects the H_2_ Rabi splitting. Results are shown for the multimode JC Hamiltonian, QED-FCI, and the effective mode approach for QED-CC. We notice that, as expected, the single-mode approximation underestimates the Rabi splitting by almost a factor of two. All the multimode methods therefore show a large improvement once the second mode has been added. Inclusion of additional modes still enlarges the splitting, although we note that the change is quite small when compared to the improvement observed adding the second plasmon in the picture. Despite using a single bosonic operator, the effective mode QED-CC allows us to almost exactly capture the multimode effect with a predicted Rabi splitting of 40.49 meV compared to 41.09 meV (QED-FCI value). We notice that the QED-FCI and JC results are not exactly equal and that the error increases when more modes are considered. This difference is due to the relaxation of the electronic ground and excited states induced by the presence of the nanoparticle. This effect is not captured unless an *ab initio* approach is used. If the electronic wave function is not optimized in the QED-FCI calculations, thus not accounting for the mutual polarization with the NP (the no corr. QED-FCI in Figure 3), the difference between QED-FCI and JC is dramatically reduced. Nonetheless, the differences between the *ab initio* method and the two-level approximation are small when compared to the improvement from 1 to 2 modes.

**Figure 3:**
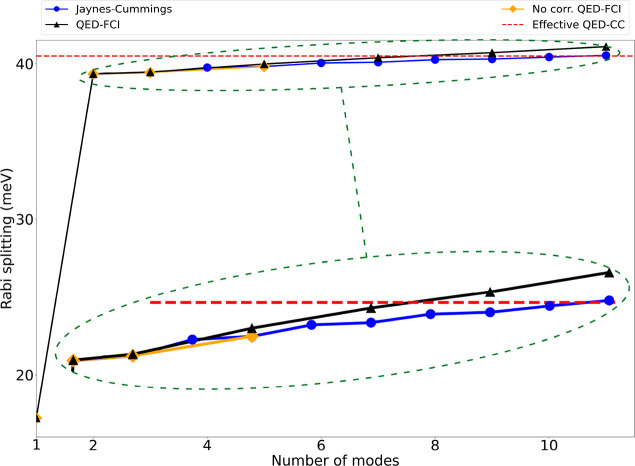
Computed Rabi splitting for the setup shown in Figure 1 as a function of the number of plasmonic modes included in the Hamiltonian. The calculations have been perfomed with the following methods: multimode Jaynes–Cummings, QED-FCI, and QED-FCI without relaxation of the electronic wave function (no corr. QED-FCI). We highlight that the latter curve is basically overlapped with the blue one. The effective mode QED-CC (dashed red line) recovers most of the multiphoton contribution. We note that the effective mode optimization has been computed using the first 12 plasmon modes of the nanoparticle setup with nonzero coupling with the molecular transition, starting from the low-energy modes. In order to contribute significantly to the splitting, the modes need to both couple with the electronic transition and be close in energy to the molecular excitation. Only some of the relevant modes are bright (such as those shown in Figure 2a,b); that is, they have a nonzero transition dipole moment. Others are dark, even though their coupling with the molecule is sizable.

We also investigated other excited-state properties, like the molecular contribution to the transition dipole moment in the GS → LP/UP transition. As shown in Figure 4a,b, the ratio between the molecular *y* and *z* components (the H_2_ molecule lies in the *yz* plane) of the polaritonic transition dipole approaches the exact QED-FCI limit when the effective mode is used. On the other hand, the agreement is significantly worse using either mode 1 or mode 2 separately. This shows that the effective mode not only improves the Rabi splitting description compared to the single-mode approximation but also provides a better description of the most important excited state properties, e.g., transition dipoles/densities.

**Figure 4:**
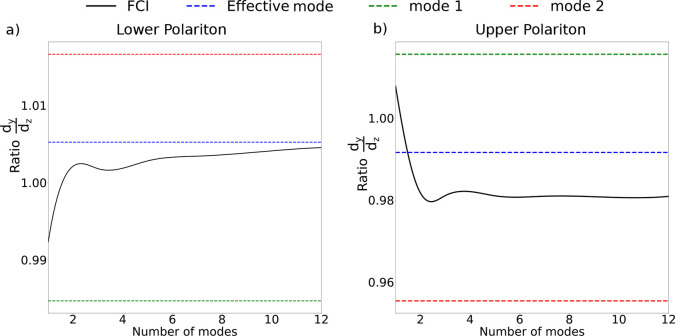
Ratio of the *y* and *z* components of the LP (a)/UP (b) transition dipole for the setup of Figure 1 in 4 different cases: QED-CC using mode 1 (dashed green line) or mode 2 only (dashed red line), effective mode QED-CC (dashed blue line), and QED-FCI (solid black curve). The QED-FCI data are reported as a function of the number of plasmonic modes included in the Hamiltonian, up to 12, which corresponds to the maximum number of modes employed in the effective mode optimization.

The qualitative picture does not change if a more complicated molecule like paranitroaniline (PNA) is placed between the three nanoellipsoidal structures (see Figure S6 of the SI). Comparing the multimode JC results with the effective mode approach for PNA, we notice that, similarly to the H_2_ case, the effective mode QED-CC recovers most of the multimode contribution. Specifically, the Rabi splitting predicted by QED-CC is almost the same as using 5 field modes in the JC approach (78.3 meV). In Figure 5, we compare the GS → LP transition densities of PNA when either the effective mode or a single-mode approach is used. Notably, an enhancement of the LP charge transfer character can be observed moving from the single-mode QED-CC with the lowest plasmon mode ( of Figure 2a) to the effective mode QED-CC, . The difference between the two transition densities, , indeed shows an increased negative density contribution on the NO_2_ group (acceptor) and an increased positive density contribution on the NH_2_ group (donor). The opposite trend is observed in the case of mode 2 (Figure 2b), meaning that in the mode 2 case more charge is transferred compared to the effective mode case. These findings can easily be rationalized using the theory described above. Indeed, since mode 2 favors the charge separation more than mode 1, the effective plasmon, that is, a linear combinations of mode 1 and mode 2, predicts an intermediate transfer between the two. Since an increasing number of modes are coupled with the main molecular transition, nanoplasmonic systems with multiple almost degenerate excitations represent a promising option to increase the field effects without reducing the field quantization volume.

**Figure 5:**
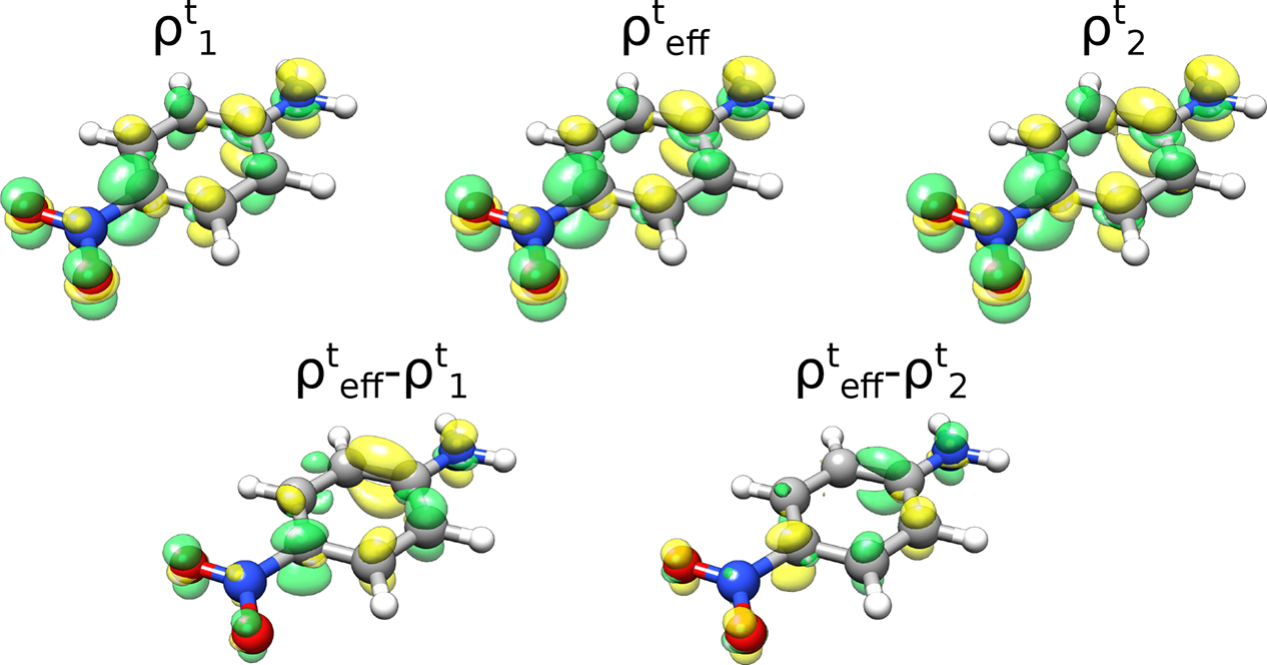
PNA transition density plots for the GS → LP transition (see setup of Figure S6 in the SI), computed using QED-CC with modes 1 and 2 or the effective mode. Positive density contributions are reported in yellow, whereas negative ones are reported in green. The difference between the transition density obtained with the effective mode and either mode 1 or mode 2 is also shown, thus allowing an easier visualization of the major changes in the PNA transition density upon changing the plasmonic part of the QED-CC Hamiltonian.

To conclude, building on the previously developed Q-PCM-NP/QED-CC model,^63^ we propose here a framework to account for multimode environments using a single effective mode. Our approach captures the main features arising from the simultaneous coupling to multiple plasmons while retaining the same computational cost of single-mode methods.^54,63^ Physical quantities, such as Rabi splittings and transition dipoles, are correctly reproduced, as verified by benchmarking against exact multimode QED-FCI for the hydrogen molecule surrounded by 3 ellipsoidal nanoparticles. The same theoretical approach is applied to a larger organic molecule, *para*-nitroaniline (PNA), where QED-FCI or multimode calculations are out of reach. Our results demonstrate that the inclusion of multiple modes is critical to correctly evaluate the plasmon–matter interaction in the case of quasi-degenerate plasmonic modes. In these cases, indeed, the single-mode approximation naturally breaks down. We notice that the effective mode scheme can be applied to any kind of wave function approximation and is not specific for plasmonic systems. We also point out that the effective mode is optimized to correctly reproduce the upper and lower polaritons only. Therefore, no improvement in the description of the ground state should be expected with this methodology. A generalization of the method should, however, be able to model the effect of multiple plasmonic modes on the molecular ground state. This topic will be the subject of a future publication. As a number of *ab initio* QED methods have started to appear recently,^3,52−57,59,72^ the here-developed effective mode approach will be of great use in all those cases where multimode effects need to be taken into account, while retaining a computationally feasible methodology.^44−48^

## Supporting Information Available

Computational details and additional numerical tests regarding Rabi splitting convergence; effect of NP shape and molecule location on the effective mode scheme; transition dipole asymptotic behavior of Figure 4; weak-coupling regime; analysis of the PNA rabi splitting similar to Figure 3 (PDF)
