# Supplementary material for: Effective Single-Mode Methodology for Strongly Coupled Multimode Molecular-Plasmon Nanosystems
Source: Nano Lett. 2023 May 23;23(11):4938–46. doi: 10.1021/acs.nanolett.3c00735 (PMC10273460; doi:10.1021/acs.nanolett.3c00735)
Supplement: Supplementary file 1 — nl3c00735_si_001.pdf [file nl3c00735_si_001.pdf]

Supporting information: Effective single-mode methodology for strongly coupled multimode molecular-plasmon nanosystems

Marco Romanelli,[†,#] Rosario Roberto Riso,[‡,#] Tor S. Haugland,[‡] Enrico Ronca,[¶] Stefano Corni,[*,†,§,∥] and Henrik Koch[*,‡,⊥]

†*Department of Chemical Sciences, University of Padova, via Marzolo 1, 35131 Padova, Italy*

‡*Department of Chemistry, Norwegian University of Science and Technology, 7491 Trondheim, Norway*

¶*Department of Chemistry, Biology and Biotechnology, University of Perugia, Via Elce di Sotto, 8, 06123, Perugia, Italy*

§*CNR Institute of Nanoscience, via Campi 213/A, 41125 Modena, Italy*

∥*Padua Quantum Technologies Research Center, University of Padova, 35131 Padova, Italy*

⊥*Scuola Normale Superiore, Piazza dei Cavalieri 7, 56126 Pisa, Italy*

#*These authors equally contributed to the work*

E-mail: stefano.corni@unipd.it; henrik.koch@sns.it

1 Additional numerical tests on the H_2 case

1.1 Numerical convergence of Rabi splitting

We checked whether results reported in Fig.3 (main text) are converged with respect to the number of modes included in the Hamiltonian. Notably, the molecule-mode coupling value decreases as the mode order increases (see Fig.S1a). This is due to the fact that higher-order modes feature charge distributions that vary so rapidly over space that the molecule does not feel any net electric field. Consequently, the corresponding Rabi splitting including more and more modes rapidly converge (see Fig.S1b).

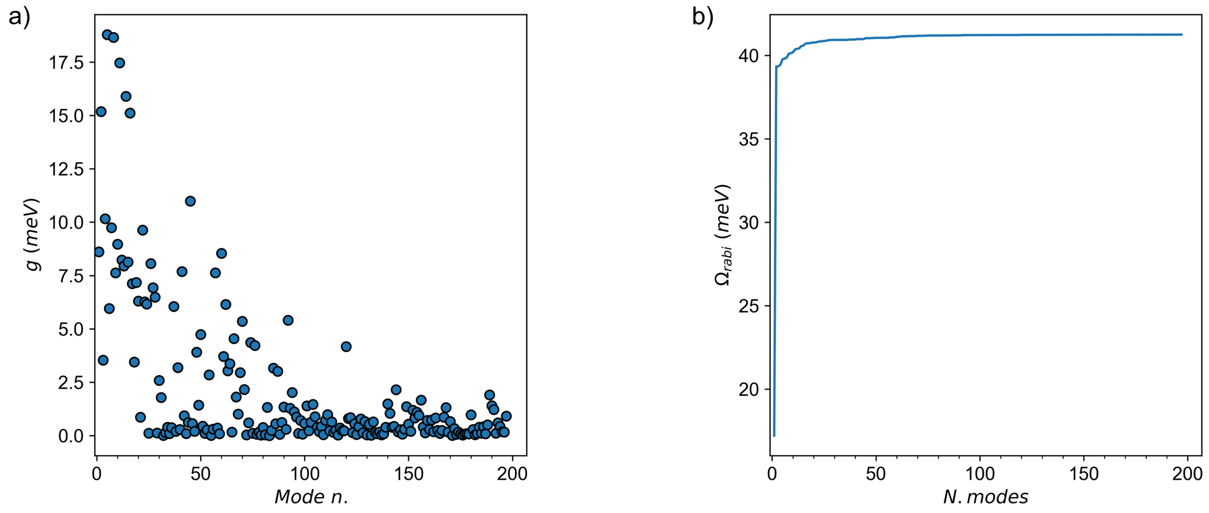

Figure S1: a) Computed coupling between the H_2 molecular transition and a given mode n. The progression goes from low-order mode, such as dipolar ones, up to high-order multipolar modes. b) Computed Jaynes-Cummings Rabi splitting as a function of the number of plasmonic modes included in the Hamiltonian, whose correspoding coupling with the molecule is reported in panel a).

1.2 Effect of NPs shape on Rabi splitting and effective mode

In this section we tested the performance of the effective mode approach on a setup similar to the one used in Fig.1 (main text) except for the aspect ratios (AR) of the ellipsoidal nanoparticles. In particular, an AR decrease (Fig.S2) leads to the well-know blue-shift of

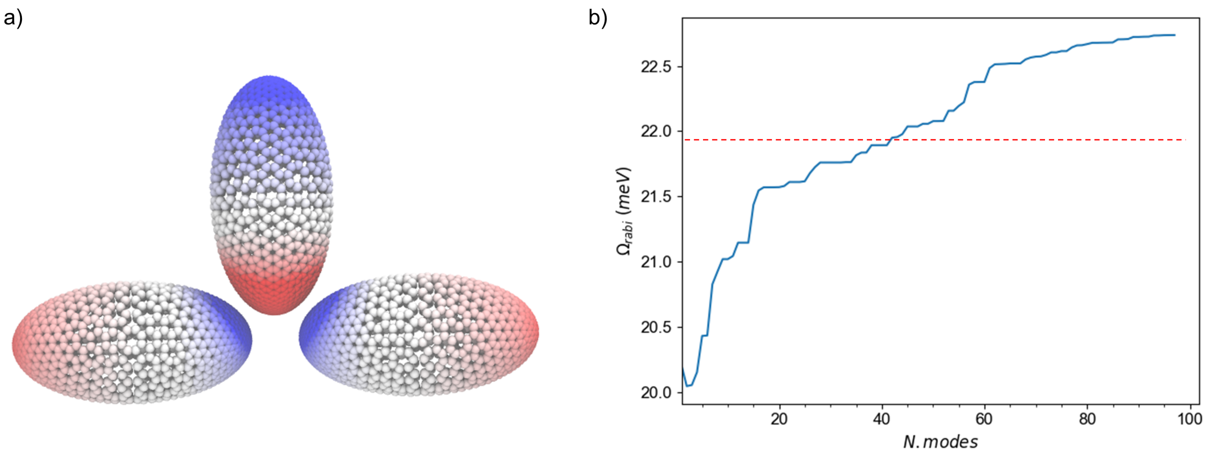

Figure S2: a) Lowest plasmon mode obtained with three ellipsoidal NPs arranged as in Fig.1 (main text), but having a smaller aspect ratio ($AR = 2$). Each NP of Fig.1 (main text) features an aspect ratio of $AR = 3$. The decrease of AR leads to a blue-shift of all modes frequencies, in particular the lowest mode shown now lies $\approx$ 200 meV above the H_2 molecular transition. b) Jaynes-Cummings rabi splitting as a function of n. of modes included in the Hamiltonian (blue line) obtained after shifting all modes frequencies of system a) such that the lowest mode gets in resonance with the H_2 transition. The horizontal steps in the rabi splitting trend comes from modes that do not couple to the molecule, thus not contributing to the polaritonic splitting. The red-dashed line is the corresponding QED-CC effective mode result.

the corresponding dipolar plasmon modes, leading to a situation where the H_2 molecular transition is non-resonant with the plasmonic modes.

If all modes frequencies obtained with the setup of Fig.S2a are shifted such that the lowest mode gets in resonance with the H_2 molecular transition, we end up with a situation where a proper Rabi splitting can be measured (Fig.S2b). However, in this case higher-energy modes do not add a significant contribution to the Rabi splitting (indeed in going from $1 \rightarrow 100$ modes the corresponding rabi splitting changes by just $\approx$ 3 meV). The reason for this is that the AR decrease not only induces a blue-shift of modes frequencies, but also increase the mode-mode energy separation. Only the lowest plasmonic mode, therefore, is in resonance with the molecular transition and significantly contributes to the splitting. The others modes are too high in energy to provide sizeable mixing.

1.3 Transition dipole asymptotic behaviour

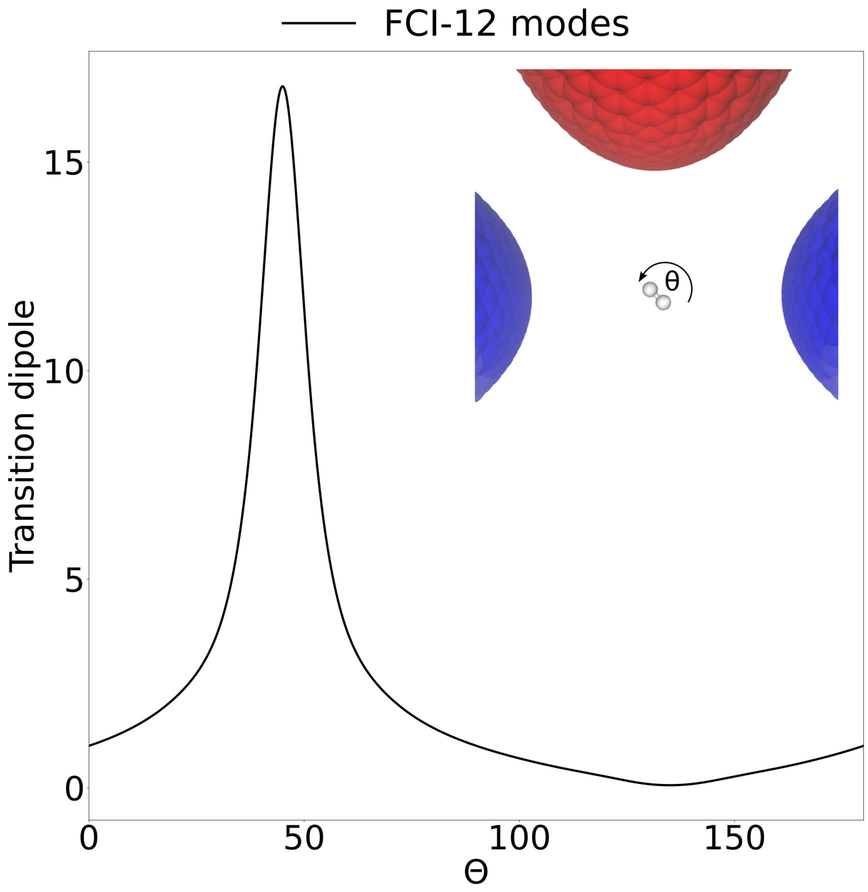

Figure S3: Dispersion with respect to the hydrogen orientation of the $\frac{d_y}{d_z}$ ratio for the lower polariton in the setup of Figs. 1-3 main text. $\Theta = 0$ corresponds to the initial geometry of Fig.1 where the H_2 molecule is inclined at 45°.

In Fig.4 of the main text, we display how the ratio between the y and z components of the transition dipole is affected by the multimode description. In particular, we observe that when the H_2 molecule is inclined at 45°, the $\frac{d_y}{d_z}$ ratio at the QED-FCI level is a bit above one for the LP and a bit lower than one for the UP. Every mode changes the value of the $\frac{d_y}{d_z}$ ratio (some significantly, some to a lesser extent). This is because, while in vacuum $\frac{d_y}{d_z}$ for the H_2 molecule is exactly equal to one, every new mode adds some spacial anisotropy differentiating between the y and z directions. Since in the strong coupling regime the

plasmons influence the molecular structure, the setup anisotropy is reflected in the behaviour of the electronic property. The aforementioned nanoparticle anisotropy is easily visualized from the two main modes reported in Fig.2 of the main text, whose transition dipoles lie on y and on z respectively. The dispersion of $\frac{d_y}{d_z}$ with respect to the hydrogen orientation is shown in Fig.S3. All significant modes have been included in the QED-FCI calculations and only the lower polariton dispersion has been plotted, as the upper polariton shows the same qualitative behaviour. We observe that by and large the geometrical features remain prevalent, with a maximum when the molecule is oriented on y (45°) and a minimum when the H-H bond is oriented along z (135°).

1.4 Different molecule spatial location

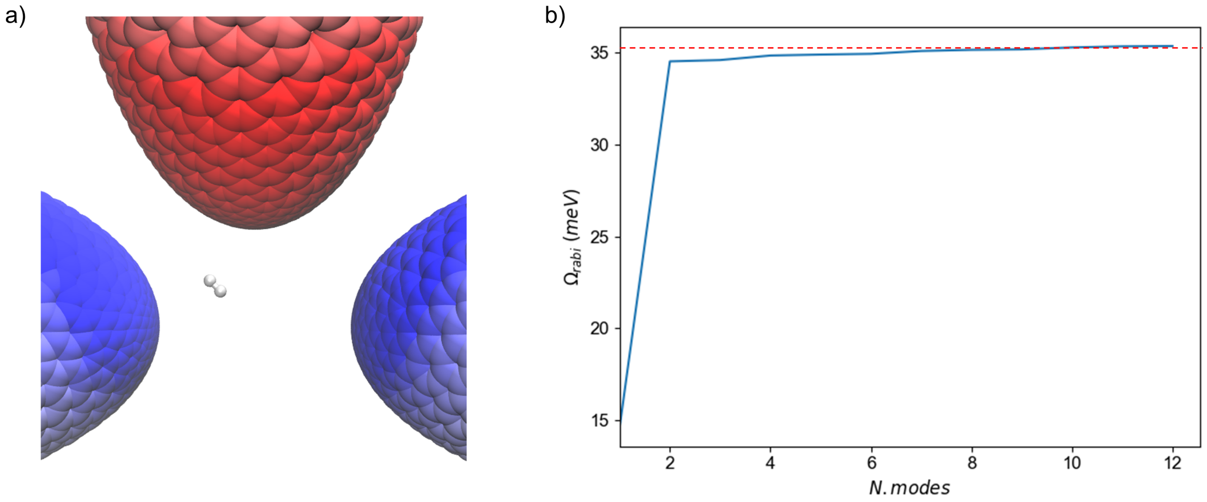

Figure S4: a)Same NPs setup shown in Fig.1 main text, but with the H_2 molecule shifted closer to two NPs edges, away from the center. b) Multimode JC Rabi splitting as a function of the number of modes included in the Hamiltonian (blue curve) obtained with setup shown in panel a) and corresponding QED-CC effective mode results (red dashed line). Only modes significantly coupled to the molecular transition have been considered, as in Fig.3 main text.

Analogous calculations to those shown in main text Figs.1-3 have been repeated with same NPs setup but different molecule location, i.e. by displacing the H_2 molecule closer to two NPs edges, see Fig.S4a. Both the multimode Jaynes-Cummings and QED-effective

mode results (Fig.S4) do not change qualitatively compared to what is shown in Fig.3 (main text). We just note that the Rabi splitting observed with such a molecular displacement is smaller compared to the case where the H_2 is placed exactly at center of the three NPs (main text, Figs. 1-3).

1.5 Weak coupling regime

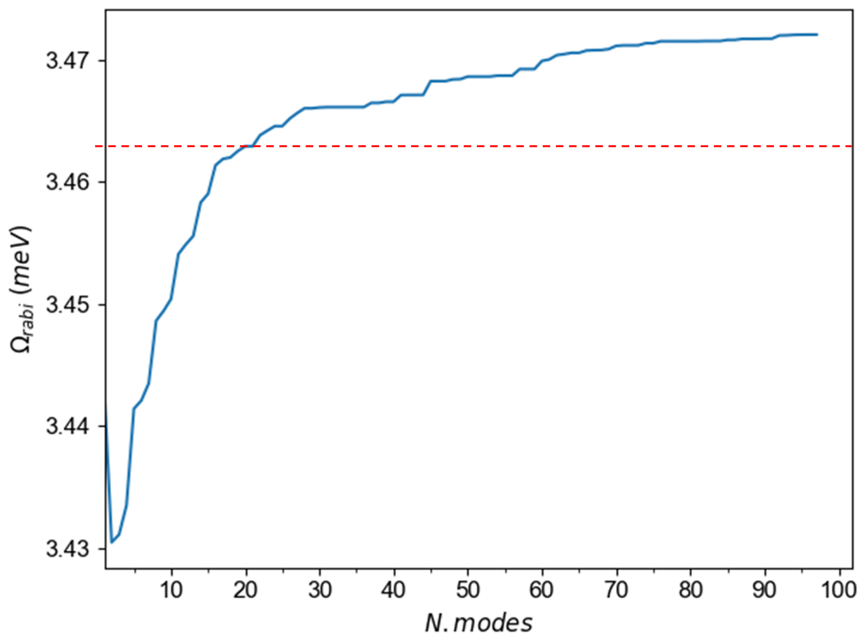

Figure S5: JC Rabi splitting as a function of the number of modes included in the Hamiltonian obtained using the same setup of Fig.1 (main text), but scaling the corresponding modes-molecule coupling values to be in the weak coupling regime. The red-dashed line is the effective mode result.

Based on the setup shown in Fig.1 (main text), we additionally test our effective mode scheme in the weak coupling limit, that has been artificially obtained by scaling the corresponding mode-molecule coupling values by 1/5 (physically speaking this screening effect could be induced by enlarging the NPs-molecule distance, for instance). The results are shown in Fig.S5, which clearly represent a typical weak coupling situation since the maximum Rabi splitting achieved is in the order of ≈ 3.5 meV, thus making it practically

negligible. Besides, we note that in this case the effective mode correction, although improving over the one mode case, is not essential. Indeed, going from $1 \rightarrow 100$ modes, the predicted Rabi splitting only changes by $\approx 0.04\ meV$. In this situation, a semiclassical description would be enough to properly describe purely molecular properties (such as excitation energies and excited state decay rates) influenced by the presence of the plasmons.[1] Moreover, a semiclassical approach would also allow for the explicit inclusion of all mode effects, since they are intrinsically embodied in the classical response function.[1,2]

2 PNA Rabi splitting: multimode effects

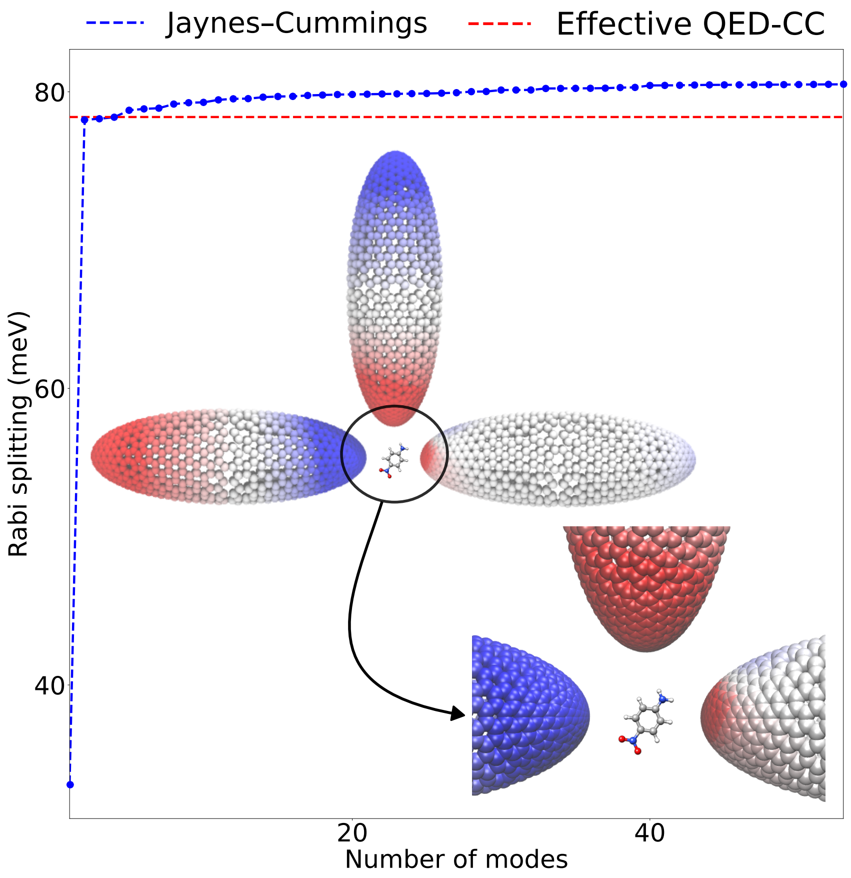

Figure S6: Computed Rabi splitting for a setup of 3 ellipsoidal NPs surrounding a PNA molecule. The average distance from the metallic surfaces is ≈ 0.6 nm (setup shown as inset). The dashed blue line are the results obtained through the multimode Jaynes-Cummings Hamiltonian (see Eq.5, main text), whereas the red one is the outcome of the QED-CC effective mode approach. Visualization of the optimized effective mode is shown in the inset.

3 Computational details

We first tested the effective mode methodology for H_2 in order to make direct comparisons with QED-FCI benchmark calculations (larger systems would not have been computationally feasible). The surface meshes were created using the Gmsh code[3] and consist of ≈ 4500

tesserae. The Drude-Lorentz parameters used to define the metal dielectric function are $\Omega_P = 8.605$ eV, $\gamma = 0.217$ eV and $\omega_0 = 12.517$ eV, which is very close to the value adopted for silver in previous literature works.[4] The damping rate and the natural frequency of the oscillator were chosen such that the NPs lowest plasmon mode is resonant with the H_2 $S_0 \rightarrow S_1$ molecular transition, around 12.7 eV according to CCSD/aug-cc-pVDZ calculations in vacuum.[5–7] In order to avoid fictitious charge transfer effects between the nanoparticles, an *a posteriori* charge normalization scheme, described in Ref.,[8] has been applied to each NP.

A similar setup was used for testing the effective mode performance with the PNA molecule since it features a richer molecular structure and is relevant for practical applications.[9,10] This time ω_0 was set to 4.354 eV such that the first plasmonic modes match the first transition of PNA at approximately 4.8 eV, according to CCSD/cc-pVDZ.[5–7] The electronic calculations were performed using a development version of the eT program.[11]

References

(1) Fregoni, J.; Haugland, T. S.; Pipolo, S.; Giovannini, T.; Koch, H.; Corni, S. Strong Coupling between Localized Surface Plasmons and Molecules by Coupled Cluster Theory. *Nano Lett.* **2021**, *21*, 6664–6670.

(2) Corni, S.; Pipolo, S.; Cammi, R. Equation of Motion for the Solvent Polarization Apparent Charges in the Polarizable Continuum Model: Application to Real-Time TDDFT. *J. Phys. Chem. A* **2015**, *119*, 5405–5416.

(3) Geuzaine, C.; Remacle, J.-F. Gmsh: A 3-D finite element mesh generator with built-in pre- and post-processing facilities. *Int. J. Numer. Meth. Eng.* **2009**, *79*, 1309–1331.

(4) Zeman, E. J.; Schatz, G. C. An accurate electromagnetic theory study of surface enhancement factors for silver, gold, copper, lithium, sodium, aluminum, gallium, indium, zinc, and cadmium. *J. Phys. Chem.* **1987**, *91*, 634–643.

(5) Pritchard, B. P.; Altarawy, D.; Didier, B.; Gibsom, T. D.; Windus, T. L. A New Basis Set Exchange: An Open, Up-to-date Resource for the Molecular Sciences Community. *J. Chem. Inf. Model.* **2019**, *59*, 4814–4820.

(6) Dunning, T. H. Gaussian basis sets for use in correlated molecular calculations. I. The atoms boron through neon and hydrogen. *J. Chem. Phys.* **1989**, *90*, 1007–1023.

(7) Kendall, R. A.; Dunning, T. H.; Harrison, R. J. Electron affinities of the first-row atoms revisited. Systematic basis sets and wave functions. *J. Chem. Phys.* **1992**, *96*, 6796–6806.

(8) Romanelli, M.; Dall'Osto, G.; Corni, S. Role of metal-nanostructure features on tip-enhanced photoluminescence of single molecules. *J. Chem. Phys.* **2021**, *155*, 214304.

(9) Alturki, A. A.; Alharbi, A. F.; Zoromba, M. S.; Abdel-Aziz, M.; Al-Hossainy, A. Poly-

meric solar cell with 18.06poly(para-nitroaniline)/TiO2 composites. *Opt.* **2023**, *136*, 113502.

(10) Ito, S.; Chen, P.; Comte, P.; Nazeeruddin, M. K.; Liska, P.; Péchy, P.; Grätzel, M. Fabrication of screen-printing pastes from TiO2 powders for dye-sensitised solar cells. *Prog. Photovolt.* **2007**, *15*, 603–612.

(11) Folkestad, S. D.; Kjønstad, E. F.; Myhre, R. H.; Andersen, J. H.; Balbi, A.; Coriani, S.; Giovannini, T.; Goletto, L.; Haugland, T. S.; Hutcheson, A., et al. e T 1.0: An open source electronic structure program with emphasis on coupled cluster and multilevel methods. *J. Chem. Phys.* **2020**, *152*, 184103.
